# Multiple Simultaneous Ranging in IR-UWB Networks

**DOI:** 10.3390/s19245415

**Published:** 2019-12-09

**Authors:** Shashi Shah, Tanee Demeechai

**Affiliations:** 1 National Science and Technology Development Agency, Phahonyothin Road, Pathumthani 12120, Thailand; shashi.sha@ncr.nstda.or.th; 2 National Electronics and Computer Technology Center, Phahonyothin Road, Pathumthani 12120, Thailand

**Keywords:** ranging, time-of-flight, clock offset, air time occupancy, ultra-wideband, two-way ranging

## Abstract

Growth in the applications of wireless devices and the need for seamless solutions to location-based services has motivated extensive research efforts to address wireless indoor localization networks. Existing works provide range-based localization using ultra-wideband technology, focusing on reducing the inaccuracy in range estimation due to clock offsets between different devices. This is generally achieved via signal message exchange between devices, which can lead to network congestion when the number of users is large. To address the problem of range estimation with limited signal messages, this paper proposes multiple simultaneous ranging methods based on a property of time difference of reception of two packets transmitted from different sources in impulse-radio ultra-wideband (IR-UWB) networks. The proposed method maintains similar robustness to the clock offsets while significantly reducing the air time occupancy when compared with the best existing ranging methods. Experimental evaluation of ranging in a line-of-sight environment shows that the proposed method enables accurate ranging with minimal air time occupancy.

## 1. Introduction

With an advancement in ubiquitous access to wireless technology and the large-scale proliferation of wireless devices with communication capabilities, the need for real-time localization of portable devices has enabled a wide range of context-aware applications and services in several industries, health sector, surveillance, disaster management, academia, and so on [1,2,3]. Real-time location systems (RTLSs) for user-centric location-based services are gaining significant attention as both service providers and end users can benefit from them. For example, they may be used to monitor and navigate inventories in a warehouse, or provide context-aware services within proximity such as to assist patients with health-care facilities in hospitals or customers in shopping malls.

The global positioning system (GPS)-based RTLSs [4,5] are used in a wide range of applications, commonly in our daily life for automotive navigation, as they provide global coverage and precision to an area of 1–5 m. However, their applicability is mostly limited to outdoor RTLSs, since they require line-of-sight with GPS satellites [1,5]. In contrast, indoor RTLS applications must inherit stringent requirements such as high localization accuracy, robustness to noise, interference, and wireless multi-path effects, and so on [1,2,3]. In this regard, the very short time-domain pulses of IR-UWB systems make them outstanding techniques commonly used by researchers and industry in various fields [3]. IR-UWB systems are well known for their capability of providing very high accuracy in scenarios satisfying line-of-sight conditions, although complex techniques are needed if this is not the case in order to obtain accurate results [3]. This capability was introduced and supported in IEEE 802.15.4a as UWB physical layer (PHY) [6,7], and later integrated in the IEEE 802.15.4 standard as UWB PHY [8].

In IR-UWB RTLSs, the location of a mobile node is computed from range-based measures relative to other anchor nodes in its vicinity. Two commonly used measures are time of flight (TOF) and time difference of arrival (TDOA), as they make use of the fact that an UWB signal has high time resolution to increase the localization accuracy relative to other techniques [9]. The TOF between a mobile and an anchor node is usually estimated by a two-way ranging (TWR) method, which typically relies on exchange of signal messages between the two nodes. While a location computation requires several TOF values, the required massive exchange of signal messages over the air tends to limit the number of serviceable mobile nodes in the RTLS. The TDOA method is an alternative to the TWR method, respecting a consideration to limit the air time occupancy required to obtain the measures for localization. This is achieved since a mobile node may transmit only a single packet over the air in order to estimate several TDOA values. However, clock offsets exist between anchor nodes due to imperfections of clock oscillators in physical environments [10,11]. Therefore, to obtain accurate results, TDOA estimation needs to deal with the clock offsets by a network-wide arrangement, which is practically hard to realize in large-scale wireless systems [12,13]. In this regard, we have found that the asynchronous TDOA method of [14] emerges as a cost-effective solution, as it uses only UWB timestamp information to effectively mitigate the clock offsets without clock synchronization between anchor nodes.

Clock offsets between mobile and anchor nodes also affect the accuracy of the TWR method. The single-sided TWR (SS-TWR) [8], symmetric double-sided TWR (SDS-TWR) [8], alternative double-sided TWR (AltDS-TWR) [15], and asymmetric double-sided TWR (ADS-TWR) [16] are considered as main TWR methods from which other available TWR methods in the literature are mainly derived [17]. The SS-TWR and SDS-TWR methods are asynchronous ranging methods introduced by the IEEE 802.15.4 UWB PHY [8] as alternatives to one-way ranging, which requires clock synchronization between network nodes. The SS-TWR employs two messages, starting with a poll message from a first node, and followed by a response message from a second node. The TOF is computed from round-trip time, from poll message marker sent to response message marker received, measured by the first node, and reply-delay time, from poll message marker received to response message marker sent, measured by the second node. This method severely suffers from clock offset between the two nodes, as the estimation error is approximately proportional to the reply-delay time, which is inevitably larger than the poll message duration. Aiming at mitigating the clock offset, the other three TWR methods provide that the first node also sends another message as a reply to the response message from the second node, to create a second side of round-trip and reply-delay times as additional information to estimate the TOF. However, to effectively mitigate the clock offset, the SDS-TWR method of estimation requires that the two reply-delay times are the same. Also, the ADS-TWR method requires that the last reply-delay time is zero, which is not feasible practically. The AltDS-TWR does not have any restrictions on the reply-delay time, which is preferable in typical systems where mobile nodes also carry variable-length sensing data. Based on a recent work [17], where state-of-the-art TWR methods have been studied respecting their estimation performance, the AltDS-TWR method is most preferred in real-world scenarios due to its robustness against variation of reply-delay time and clock offsets. Interestingly, the alternative double-sided TWR with passive ranging (AltDS-TWR&PR) [18] method has been proposed recently as an improvement over the symmetric double-sided TWR with passive ranging (SDS-TWR&PR) [19] method, where the TOFs from a mobile node to several anchor nodes can be obtained simultaneously with a minimal number of packets over the air. However, the method requires that a ranging session can only be initiated by an anchor node and lacks flexibility in implementation for a variety of RTLS applications.

In this paper, we address the problem of range estimation in IR-UWB RTLSs, where the air time occupancy is minimized and the clock offset problem is properly handled. In this regard, a multiple simultaneous ranging method is proposed, which is a novel extension of TWR methods. In this method, the TOFs from a mobile node to several anchor nodes are obtained simultaneously with a minimal number of packets over the air, while the clock offset problem is handled similarly to [14,15]. We also show that the TOF obtained by [15] is equivalent to the TOF obtainable by the proposed method, while the TDOA obtained by [14] is equivalent to the TDOA obtainable by the proposed method. The formulation of the proposed method provides a clear insight into the ranging problem, so that we can implement it by a variety of schemes. Estimation performance is also studied by analysis as well as experiment that compares the method with AltDS-TWR and AltDS-TWR&PR methods.

## 2. Multiple Simultaneous Ranging

### 2.1. Proposed Method

Suppose a wireless network comprises anchor nodes, denoted by A, B, and C, placed at fixed positions, and a mobile node, denoted by M, placed at unknown position. Each node employs a UWB signal with nanosecond accuracy for sample-based timestamps. Hence, based on its own clock, each node can determine the transmission or reception timestamp value of a transmitted or received packet with nanosecond accuracy. Also, it can align the transmission time of a packet with a pre-specified timestamp value.

The proposed ranging method originates from analyzing a property of the time difference of reception of two packets transmitted from different sources. Assume without loss of generality that nodes M and A transmit the first and second packets respectively. Denote the time difference of reception at node *X* as $P_{X}$. Then, for any receptor nodes *X* and *Y*, it can be shown that

(1) $$P_{X}-P_{Y}=\left(T_{P(A,X)}-T_{P(A,Y)}\right)-\left(T_{P(M,X)}-T_{P(M,Y)}\right),$$

where $T_{P(X,Y)}$ denotes the TOF from node *X* to node *Y*. It is straightforward to derive Equation (1) by directly modeling a packet-reception time instant as summation of packet-transmission time instant, transmitting analog delay of the transmitter node, TOF from the transmitter node to the receptor node, and receiving analog delay of the receptor node.

In the proposed method, the clock of a particular node is selected as a preferred clock, and the time difference of reception for each node within the common coverage of the two packets is determined as if it was measured by the preferred clock. Denote the selected node of preferred clock as node *W*. Hence, for nodes M, A, B, and C, the corresponding time difference of reception can be computed by

(2) $${\hat{P}}_{M}=\left\{t_{\mathrm{Rx}(2)}^{M}-\left(t_{\mathrm{Tx}(1)}^{M}+d_{M}\right)\right\}r_{M}^{W},$$

(3) $${\hat{P}}_{A}=\left\{\left(t_{\mathrm{Tx}(2)}^{A}+d_{A}\right)-t_{\mathrm{Rx}(1)}^{A}\right\}r_{A}^{W},$$

(4) $${\hat{P}}_{B}=\left\{t_{\mathrm{Rx}(2)}^{B}-t_{\mathrm{Rx}(1)}^{B}\right\}r_{B}^{W},$$

and

(5) $${\hat{P}}_{C}=\left\{t_{\mathrm{Rx}(2)}^{C}-t_{\mathrm{Rx}(1)}^{C}\right\}r_{C}^{W},$$

where $r_{X}^{Y}$ denotes the ratio of node-*Y* clock speed over node-*X* clock speed—estimation of the numerical value will be discussed herein, $d_{X}$ denotes summation of transmitting and receiving analog delays of node *X*, and $t_{\mathrm{Tx}(n)}^{X}$ and $t_{\mathrm{Rx}(n)}^{X}$ denote respectively the transmission and reception timestamp values read by node *X* for the *n*-th packet—recall that the first and second packets are transmitted by nodes M and A respectively. While the value of $d_{X}$ should be obtained as if it was measured with the node-*X* clock for Equations (2) and (3) to be exactly correct, the error would still be negligible if it was obtained before the ranging operation by a measurement with another typical crystal clock. It should be noted also that Equations (2)–(5) are given for the case that both transmission and reception timestamps have not been calibrated respecting the transmitting and receiving analog delays. However, in the case that the timestamps have been all perfectly calibrated respecting the analog delays, Equations (2) and (3) become

(6) $${\hat{P}}_{M}=\left\{t_{\mathrm{Rx}(2)}^{M}-t_{\mathrm{Tx}(1)}^{M}\right\}r_{M}^{W},$$

(7) $${\hat{P}}_{A}=\left\{t_{\mathrm{Tx}(2)}^{A}-t_{\mathrm{Rx}(1)}^{A}\right\}r_{A}^{W},$$

while Equations (4) and (5) remain unchanged.

In order to determine $r_{X}^{W}$, a method is provided such that one of the two transmitter nodes is selected to transmit another packet (the third packet) with a pre-specified transmission time difference between the two packets transmitted by itself. Denote this transmission time difference as δ. Assume the mobile node movement during the δ duration is negligible. Now, if the selected transmitter node is node M, then

(8) $$r_{X}^{W}=r_{M}^{W}/r_{M}^{X},$$

where

(9) $$r_{M}^{Y}=\left\{\begin{array}{cc} 1, & \mathrm{if}\;Y=M\\ (t_{\mathrm{Rx}(3)}^{Y}-t_{\mathrm{Rx}(1)}^{Y})/\delta , & \mathrm{otherwise}. \end{array}\right.$$

On the other hand, if the selected transmitter node is node A, then

(10) $$r_{X}^{W}=r_{A}^{W}/r_{A}^{X},$$

where

(11) $$r_{A}^{Y}=\left\{\begin{array}{cc} 1, & \mathrm{if}\;Y=A\\ (t_{\mathrm{Rx}(3)}^{Y}-t_{\mathrm{Rx}(2)}^{Y})/\delta , & \mathrm{otherwise}. \end{array}\right.$$

However, a more convenient design may require that node *W* is both the selected node of preferred clock and the transmitter of the first and third packets. In this case, where that node is node M, we obtain

(12) $$r_{X}^{W}=\left\{\begin{array}{cc} 1, & \mathrm{if}\;X=M\\ \delta /(t_{\mathrm{Rx}(3)}^{X}-t_{\mathrm{Rx}(1)}^{X}), & \mathrm{otherwise}. \end{array}\right.$$

After the time difference of reception for each node has been computed, the obtained values from all relevant nodes can be collected into a computing unit for obtaining the TOFs from the mobile node M to all the relevant anchor nodes. With *N* relevant anchor nodes, the N+1 collected numerical values may be used to construct *N* independent equations based on Equation (1). Here three equations can be constructed from $P_{M}$, $P_{A}$, $P_{B}$, and $P_{C}$ as

(13) $$P_{M}-P_{A}=\left(T_{P(A,M)}-T_{P(A,A)}\right)-\left(T_{P(M,M)}-T_{P(M,A)}\right),$$

(14) $$P_{M}-P_{B}=\left(T_{P(A,M)}-T_{P(A,B)}\right)-\left(T_{P(M,M)}-T_{P(M,B)}\right),$$

and

(15) $$P_{M}-P_{C}=\left(T_{P(A,M)}-T_{P(A,C)}\right)-\left(T_{P(M,M)}-T_{P(M,C)}\right).$$

As anchor nodes are all placed at fixed positions, the range from node A to another anchor node may be pre-measured at high accuracy, and the above three equations have only three unknown values, $T_{P(M,A)}$, $T_{P(M,B)}$, and $T_{P(M,C)}$. Accordingly, the TOF from the mobile node M to the anchor node *X* is computed by

(16) $${\hat{T}}_{P(M,X)}=\left({\hat{P}}_{M}-{\hat{P}}_{X}\right)-\frac{\left({\hat{P}}_{M}-{\hat{P}}_{A}\right)}{2}+{\hat{T}}_{P(A,X)}.$$

It may be noted from Equation (16) that the error in pre-measurement of $T_{P(A,X)}$ will add directly to the estimation error for $T_{P(M,X)}$. Therefore, it is important that the pre-measurement range error should correspond to very few centimeters to maintain UWB accuracy convention. This requirement however is much less demanding than the requirement to know all the anchor node positions accurately. The TOFs can then be simply computed from Equation (16) and multiplied with the speed of light to obtain the required ranges.

The above exemplified scheme may be denoted as MSR1, where node M transmits the first and third packets of a ranging session and is also the selected node of preferred clock (W=M). This scheme may be used in a simple RTLS, where each mobile node independently accesses the air by a pure Aloha technique, i.e., a repeated process of sleeping for a random time and then waking up and accessing the air to initiate ranging for localization. Steps of range estimation may now be presented for MSR1 as follows. Suppose node A is configured as an active anchor node which will transmit the second packet of each ranging session, and the TOFs from node A to other so-called passive anchor nodes which will listen to all the three packets have been pre-measured. Then the following steps are executed in order for ranging from node M to node A and other passive anchor nodes in their vicinity.

MSR1 (Node M is the selected node of preferred clock and the transmitter of the first and third packets):

- *Step 1:* Node M determines the transmission timestamps for the first and third packets to follow $\delta =(t_{\mathrm{Tx}(3)}^{M}-t_{\mathrm{Tx}(1)}^{M})$, based on its current timestamp value and the properly pre-specified value of δ.
- *Step 2:* Node M transmits the first packet at the determined time, while other nodes are in receiver mode.
- *Step 3:* Upon completion of the transmission and reception, all nodes except node M retrieve and store the corresponding reception timestamps. Then node A prepares for transmission of the second packet, while other nodes enter the receiver mode.
- *Step 4:* Node A transmits the second packet, while other nodes are in receiver mode.
- *Step 5:* Upon completion of the transmission and reception, node A retrieves and stores the corresponding transmission timestamp, while other nodes retrieve and store the corresponding reception timestamps. Then node M computes ${\hat{P}}_{M}$ using Equations (6) and (12), and prepares for transmission of the third packet, which will carry the value of ${\hat{P}}_{M}$, while other nodes enter the receiver mode.
- *Step 6:* Node M transmits the third packet at the determined time, while other nodes are in receiver mode.
- *Step 7:* Upon completion of the transmission and reception, all nodes except node M retrieve and store the corresponding reception timestamps. Node A also stores the received value of ${\hat{P}}_{M}$.
- *Step 8:* Node A computes ${\hat{P}}_{A}$ using Equations (7) and (12), while each passive anchor node *X* computes ${\hat{P}}_{X}$ using ${\hat{P}}_{X}=\left\{t_{\mathrm{Rx}(2)}^{X}-t_{\mathrm{Rx}(1)}^{X}\right\}r_{X}^{W}$ and Equation (12).
- *Step 9:* All anchor nodes send all the time difference of reception values, i.e., node A sends ${\hat{P}}_{A}$ and ${\hat{P}}_{M}$ while each passive anchor node *X* sends ${\hat{P}}_{X}$, to a computing unit via a communication backbone.
- *Step 10:* For each anchor node *X*, the computing unit computes ${\hat{T}}_{P(M,X)}$ using Equation (16), and then multiplies the result with the speed of light to obtain the corresponding range.

For some applications, where a time-slotted medium access technique is used for increased efficiency compared with the simple pure Aloha technique, an anchor node may necessarily send packets periodically over the air to facilitate the time slot implementation, while each mobile node acquires the slot timing by using a low-power listening method [20] on those packets. It may be beneficial that each anchor’s packet facilitating the time slot implementation also acts as the initiation packet for a ranging session. In this case, another scheme of the proposed method is more compatible. Denote the scheme as MSR2, where node A transmits the first and third packets of a ranging session and is also the selected node of preferred clock (W=A). In this case, the proposed method results in

(17) $${\hat{T}}_{P(M,X)}=\left({\hat{P}}_{X}-{\hat{P}}_{M}\right)-\frac{\left({\hat{P}}_{A}-{\hat{P}}_{M}\right)}{2}+{\hat{T}}_{P(A,X)}.$$

In addition, computation of the time difference of reception values for the mobile and active anchor nodes becomes, assuming timestamps have been all perfectly calibrated respecting the analog delays,

(18) $${\hat{P}}_{M}=\left\{t_{\mathrm{Tx}(2)}^{M}-t_{\mathrm{Rx}(1)}^{M}\right\}r_{M}^{W},$$

(19) $${\hat{P}}_{A}=\left\{t_{\mathrm{Rx}(2)}^{A}-t_{\mathrm{Tx}(1)}^{A}\right\}r_{A}^{W},$$

where

(20) $$r_{X}^{W}=\left\{\begin{array}{cc} 1, & \mathrm{if}\;X=A\\ \delta /(t_{\mathrm{Rx}(3)}^{X}-t_{\mathrm{Rx}(1)}^{X}), & \mathrm{otherwise}. \end{array}\right.$$

Then steps of range estimation with MSR2 may be presented as follows.

MSR2 (Node A is the selected node of preferred clock and the transmitter of the first and third packets):

- *Step 1:* Node A determines the transmission timestamps for the first and third packets to follow $\delta =(t_{\mathrm{Tx}(3)}^{A}-t_{\mathrm{Tx}(1)}^{A})$, based on its current timestamp value and the properly pre-specified value of δ.
- *Step 2:* Node A transmits the first packet at the determined time, while other nodes are in receiver mode.
- *Step 3:* Upon completion of the transmission and reception, all nodes except node A retrieve and store the corresponding reception timestamps. Then node M prepares for transmission of the second packet, while other nodes enter the receiver mode.
- *Step 4:* Node M transmits the second packet, while other nodes are in receiver mode.
- *Step 5:* Upon completion of the transmission and reception, node M retrieves and stores the corresponding transmission timestamp, while other nodes retrieve and store the corresponding reception timestamps. Then node A prepares for transmission of the third packet, while other nodes enter the receiver mode.
- *Step 6:* Node A transmits the third packet at the determined time, while other nodes are in receiver mode.
- *Step 7:* Upon completion of the transmission and reception, all nodes except node A retrieve and store the corresponding reception timestamps.
- *Step 8:* Node M computes ${\hat{P}}_{M}$ using Equations (18) and (20), node A computes ${\hat{P}}_{A}$ using Equations (19) and (20), while each passive anchor node *X* computes ${\hat{P}}_{X}$ using ${\hat{P}}_{X}=\left\{t_{\mathrm{Rx}(2)}^{X}-t_{\mathrm{Rx}(1)}^{X}\right\}r_{X}^{W}$ and Equation (20). Then node A enters the receiver mode.
- *Step 9:* Node M transmits a wireless data packet carrying the value of ${\hat{P}}_{M}$ to node A.
- *Step 10:* All anchor nodes send all the time difference of reception values, i.e., node A sends ${\hat{P}}_{A}$ and ${\hat{P}}_{M}$ while each passive anchor node *X* sends ${\hat{P}}_{X}$, to a computing unit via a communication backbone.
- *Step 11:* For each anchor node *X*, the computing unit computes ${\hat{T}}_{P(M,X)}$ using Equation (17), and then multiplies the result with the speed of light to obtain the corresponding range.

The clock-speed ratio $r_{X}^{W}$ may be determined by an alternative method that does not require transmission of the third packet. Note that typical receiver performs carrier-frequency offset estimation in the packet-reception process. In addition, both system clock and radio carrier signal of a node are synthesized from the same reference oscillator, and, hence, the clock-speed ratio simply equals the carrier-frequency ratio. Therefore, the clock-speed ratio may be determined from

(21) $$r_{X}^{W}=\left\{\begin{array}{cc} 1, & \mathrm{if}\;X=W\\ \left(f_{c}+\Delta f_{X}^{W}\right)/f_{c}, & \mathrm{otherwise}, \end{array}\right.$$

where $f_{c}$ is the nominal carrier frequency, and $\Delta f_{X}^{W}$ is the carrier-frequency offset estimated by the receiver of node *X* upon reception of the packet from Node *W*. Note that a commercial off-the-shelf node provides a programming interface to such carrier-frequency offset values [21], enabling utilization of Equation (21). To exploit such capability, a scheme denoted as MSR3 is proposed as an alternative to MSR2 as follows.

MSR3 (Node A is the transmitter of the first packet; No third packet; W=A):

- *Step 1:* Node A transmits the first packet, while other nodes are in receiver mode.
- *Step 2:* Upon completion of the transmission and reception, each node X,X≠A, retrieves and stores the corresponding reception timestamp, and also determines $r_{X}^{W}$ using Equation (21).
- *Step 3:* While other nodes enter the receiver mode, node M determines a suitable transmission timestamp for the second packet from the obtained reception timestamp of the first packet, and then computes ${\hat{P}}_{M}$ using Equation (18).
- *Step 4:* Node M transmits the second packet carrying the value of ${\hat{P}}_{M}$ at the determined time, while other nodes are in receiver mode.
- *Step 5:* Upon completion of the transmission and reception, each node X,X≠M, retrieves and stores the corresponding reception timestamp, and node A also stores the received value of ${\hat{P}}_{M}$.
- *Step 6:* Node A computes ${\hat{P}}_{A}$ using Equation (19) and $r_{A}^{W}=1$, while each passive anchor node *X* computes ${\hat{P}}_{X}$ using ${\hat{P}}_{X}=\left\{t_{\mathrm{Rx}(2)}^{X}-t_{\mathrm{Rx}(1)}^{X}\right\}r_{X}^{W}$.
- *Step 7:* All anchor nodes send all the time difference of reception values, i.e., node A sends ${\hat{P}}_{A}$ and ${\hat{P}}_{M}$ while each passive anchor node *X* sends ${\hat{P}}_{X}$, to a computing unit via a communication backbone.
- *Step 8:* For each anchor node *X*, the computing unit computes ${\hat{T}}_{P(M,X)}$ using Equation (17), and then multiplies the result with the speed of light to obtain the corresponding range.

### 2.2. Unification with Recent Methods

It may be shown that the formulation of the proposed method can be used to obtain the main results of recent methods of AltDS-TWR [15] and asynchronous TDOA [14]. To derive the result of [15], note that based on MSR1, the TOF between nodes M and A can be obtained from Equation (16) as

(22) $${\hat{T}}_{P(M,A)}=\left({\hat{P}}_{M}-{\hat{P}}_{A}\right)/2.$$

Assume all timestamps have been calibrated respecting the analog delays as in [15]. Then substituting Equations (6), (7), (12), and $\delta =(t_{\mathrm{Tx}(3)}^{M}-t_{\mathrm{Tx}(1)}^{M})$ into Equation (22), we obtain

(23) $${\hat{T}}_{P(M,A)}=\frac{1}{2}\left\{\left(t_{\mathrm{Rx}(2)}^{M}-t_{\mathrm{Tx}(1)}^{M}\right)-\frac{\left(t_{\mathrm{Tx}(2)}^{A}-t_{\mathrm{Rx}(1)}^{A}\right)\left(t_{\mathrm{Tx}(3)}^{M}-t_{\mathrm{Tx}(1)}^{M}\right)}{t_{\mathrm{Rx}(3)}^{A}-t_{\mathrm{Rx}(1)}^{A}}\right\}.$$

Consider nodes M and A here as nodes a and b in [15] respectively. Expressing Equation (23) using the notation of [15], we obtain

(24) $${\hat{t}}_{f}=\frac{1}{2}\left\{{\hat{R}}_{a}-\frac{{\hat{D}}_{b}\left({\hat{R}}_{a}+{\hat{D}}_{a}\right)}{{\hat{R}}_{b}+{\hat{D}}_{b}}\right\}.$$

We can then rearrange the result as

(25) $${\hat{t}}_{f}=\frac{{\hat{R}}_{a}{\hat{R}}_{b}+{\hat{D}}_{a}{\hat{D}}_{b}}{2({\hat{R}}_{b}+{\hat{D}}_{b})},$$

which is exactly same as Equation (18b) of [15], i.e., the main result of [15] when node-*A* clock is preferred.

In [14], a method to estimate the difference of TOFs (or equivalently the TDOA) from one node to any two nodes without clock synchronization has been presented. The main equation derived in [14] enabling such estimation may be expressed as

(26) $$\begin{array}{cc} {\hat{T}}_{P(S,A)}-{\hat{T}}_{P(S,B)}= & \frac{\left(t_{\mathrm{Rx}(2)}^{A}-t_{\mathrm{Rx}(1)}^{A}\right)\left(t_{\mathrm{Tx}(3)}^{R}-t_{\mathrm{Tx}(1)}^{R}\right)}{t_{\mathrm{Rx}(3)}^{A}-t_{\mathrm{Rx}(1)}^{A}}\\ \; & -\frac{\left(t_{\mathrm{Rx}(2)}^{B}-t_{\mathrm{Rx}(1)}^{B}\right)\left(t_{\mathrm{Tx}(3)}^{R}-t_{\mathrm{Tx}(1)}^{R}\right)}{t_{\mathrm{Rx}(3)}^{B}-t_{\mathrm{Rx}(1)}^{B}}\\ \; & +(T_{P(R,A)}-T_{P(R,B)}), \end{array}$$

which is obtained by combining Equations (13) and (17) of [14] and then re-expressing terms using the TOF and timestamp notations of this paper, and considering first, second, and third packets are transmitted respectively by nodes R, S, and R respectively. This equation can be used to compute the TDOA, ${\hat{T}}_{P(S,A)}-{\hat{T}}_{P(S,B)}$, when nodes R, A, and B are placed at fixed positions, and hence $T_{P(R,A)}-T_{P(R,B)}$ is known. While the mentioned state of being known is just a matter of application, it can be shown that the equation may be derived from the present formulation respecting MSR1 as follows. Note that, regarding the order of packet transmitters, the scenario of Equation (26) corresponds to the case that the nodes M, A, B, and C here respectively correspond to nodes R, S, A, and B in [14]. In addition, the following equation may be obtained either directly from Equation (1) or by subtracting Equation (14) from Equation (15):

(27) $${\hat{P}}_{B}-{\hat{P}}_{C}=\left(T_{P(A,B)}-T_{P(A,C)}\right)-\left({\hat{T}}_{P(M,B)}-{\hat{T}}_{P(M,C)}\right).$$

Then, by substituting Equations (4), (5), (12), and $\delta =(t_{\mathrm{Tx}(3)}^{M}-t_{\mathrm{Tx}(1)}^{M})$ into Equation (27), replacing M, A, B, and C by R, S, A, and B respectively, and finally rearranging terms, Equation (26) is obtained as a result.

### 2.3. Effect of Clock Offset

Consider the MSR1 scheme to represent the proposed method and the clock offset as the only source of impairments. Then the estimated TOF from node M to anchor node *X* is given according to Equation (16) by

(28) $${\hat{T}}_{P(M,X)}=\left(1+e_{M}\right)\left\{\left(P_{M}-P_{X}\right)-\frac{\left(P_{M}-P_{A}\right)}{2}\right\}+T_{P(A,X)},$$

where $e_{X}$ denotes node-*X* clock offset normalized by ideal-clock speed. Substituting

(29) $$T_{P(M,X)}=\left(P_{M}-P_{X}\right)-\frac{\left(P_{M}-P_{A}\right)}{2}+T_{P(A,X)}$$

into Equation (28) and arranging the terms, we obtain

(30) $${\hat{T}}_{P(M,X)}=T_{P(M,X)}+e_{M}\left(T_{P(M,X)}-T_{P(A,X)}\right),$$

which suggests that the error in the estimated TOF is $e_{M}\left(T_{P(M,X)}-T_{P(A,X)}\right)$. This implies that such error for the AltDS-TWR method [15] is $e_{M}T_{P(M,X)}$. Based on this result, the effect of clock offset on both methods is obviously negligible in applications with typical crystal clocks. However, such error for the proposed method will be smaller than that for the AltDS-TWR method whenever

(31) $$|T_{P(M,X)}-T_{P(A,X)}|<T_{P(M,X)},$$

or equivalently

(32) $$T_{P(M,X)}>\frac{1}{2}T_{P(A,X)}.$$

### 2.4. Effect of Timestamp Error

According to [22], the line-of-sight reception timestamps obtained by a commercial off-the-shelf node can be corrupted by systematic error, which depends on variable channel conditions. Such variation from the ideal timestamp will lead to error in the estimated range. We study the effect of such timestamp error, based on the following assumptions: (i) the mobile node remains static during a ranging session; (ii) the transmission timestamps are not corrupted by any errors; (iii) the reception timestamp error assumes the same value for all packets in both directions of the same channel defined by the positions of the two communicating nodes; and (iv) the reception timestamp errors of different channels are independent and identically distributed zero-mean random variables.

Consider the MSR1 scheme to represent the proposed method and the reception timestamp error as the only source of impairments. Then computing $r_{X}^{W}$ according to Equation (12) yields $r_{X}^{W}=1$, because the reception timestamp errors associated with $t_{\mathrm{Rx}(3)}^{X}$ and $t_{\mathrm{Rx}(1)}^{X}$ are the same. Then the estimated TOF from node M to node A can be expressed according to Equations (6) and (7), and Equation (22) by

(33) $$\begin{array}{cc} {\hat{T}}_{P(M,A)}= & \frac{\left(\left(\tau_{\mathrm{Rx}(2)}^{M}+t_{e}\left(A,M\right)\right)-t_{\mathrm{Tx}(1)}^{M}\right)-\left(t_{\mathrm{Tx}(2)}^{A}-\left(\tau_{\mathrm{Rx}(1)}^{A}+t_{e}\left(M,A\right)\right)\right)}{2}\\ = & \frac{\left(\tau_{\mathrm{Rx}(2)}^{M}-t_{\mathrm{Tx}(1)}^{M}\right)-\left(t_{\mathrm{Tx}(2)}^{A}-\tau_{\mathrm{Rx}(1)}^{A}\right)}{2}+t_{e}\left(M,A\right)\\ = & T_{P(M,A)}+t_{e}\left(M,A\right), \end{array}$$

where $\tau_{\mathrm{Rx}(n)}^{X}$ is the ideal error-free version of $t_{\mathrm{Rx}(n)}^{X}$, and $t_{e(X,Y)}=t_{e(Y,X)}$ is the reception timestamp error associated with the UWB channel between nodes *X* and *Y*. Similarly, the estimated TOF from node M to anchor node *X*, X≠A, can be expressed according to Equations (4)–(7), and Equation (16) by

(34) $${\hat{T}}_{P(M,X)}=T_{P(M,X)}+t_{e(M,X)}-t_{e(A,X)}.$$

From Equations (33) and (34) the TOF error can be expressed by

(35) $${\hat{T}}_{P(M,X)}-T_{P(M,X)}=\left\{\begin{array}{cc} t_{e(M,X)}, & \mathrm{if}\;X=A\\ t_{e(M,X)}-t_{e(A,X)}, & otherwise. \end{array}\right.$$

Accordingly, the mean square error (MSE) is

(36) $$\mathrm{MSE}\left({\hat{T}}_{P(M,X)}\right)=\left\{\begin{array}{cc} \sigma_{e}^{2}, & \mathrm{if}\;X=A\\ 2\sigma_{e}^{2}, & \mathrm{otherwise}, \end{array}\right.$$

where $\sigma_{e}$ is the standard deviation of the reception timestamp error. This implies, for the AltDS-TWR method [15], $\mathrm{MSE}\left({\hat{T}}_{P(M,X)}\right)=\sigma_{e}^{2}$. Hence, in estimating the TOFs from a mobile node to a majority of anchor nodes, the MSE of the proposed method is twice as sensitive to the reception timestamp error as that of the AltDS-TWR method. However, experimental results with *commercial of-the-shelf* nodes in Section 3.2 show that the corresponding average range error in a line-of-sight environment is still within very few centimeters.

## 3. Numerical Results

### 3.1. Air Time Occupancy

In this section, the air time occupancies of MSR1, MSR2, MSR3, AltDS-TWR, and AltDS-TWR&PR are compared in terms of the number of packets required over the air to obtain the sensing information by the fixed infrastructure. Suppose a wireless network consists of *N* number of anchor nodes, $A_{i}$, i=1,2,…,N, serving a common coverage area. Then, as detailed in Section 2.1, MSR1 takes three packets, MSR2 takes four packets, and MSR3 takes only two packets for obtaining the sensing information. Note that AltDS-TWR&PR may be executed by the same steps of wireless transmission as MSR2, since they are different only about the equation of computing the TOF. So, the method of AltDS-TWR&PR also requires four packets for obtaining the necessary sensing information.

For the analysis of AltDS-TWR, we further assume that the fixed architecture of *N* anchor nodes is known by all network nodes, including the mobile nodes. Then, in order to obtain the value of TOF from a mobile node to an anchor node, the AltDS-TWR method requires three packets, denoted as mobile-poll, anchor-response, and mobile-final (the prefix denotes the node that transmits the packet) in sequence over the air. Therefore, it would take exactly 3N packets to obtain the values of TOF to the *N* anchor nodes, since knowledge on the fixed architecture of *N* anchor nodes can be used to avoid collision. However, enabling the method of AltDS-TWR with combined transmissions for simultaneous ranging of a mobile node to several anchor nodes, it would require only N+2 packets in sequence, denoted as mobile-poll, $A_{1}$-response, $A_{2}$-response, …, $A_{N}$-response, and mobile-final. This may be achieved by provision of N+1 reserved time-slots after the mobile-poll packet has been transmitted. Notably, the air time occupancy of both the schemes of AltDS-TWR is dependent on the number of anchor nodes in a wireless network.

Table 1 summarizes the air time occupancy for obtaining the sensing information by the proposed schemes and also compares them with AltDS-TWR and AltDS-TWR&PR for a general wireless networks scenario. For the proposed schemes and AltDS-TWR&PR, the number of required packets for simultaneous ranging of a mobile node to several anchor nodes is limited and independent of the number of anchor nodes in a wireless network. As seen in Table 1, comparing the two AltDS-TWR schemes and MSR1 for the case where a mobile node is designated to be the first-packet transmitter, MSR1 is the most air efficient. In addition, comparing AltDS-TWR&PR, MSR2, and MSR3 for the case where an anchor node is designated to be the first-packet transmitter, MSR3 is the most air efficient.

### 3.2. Experimental Evaluation

**Figure 1:**
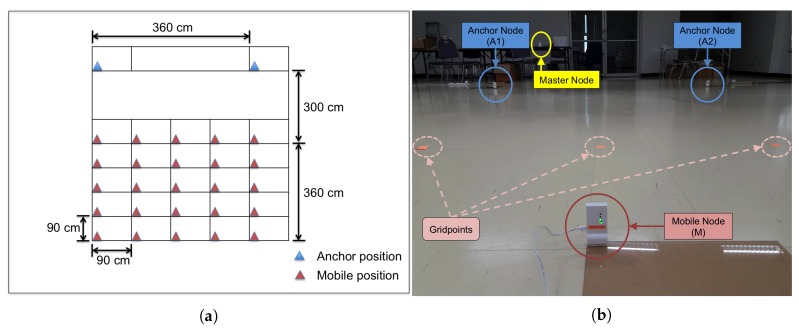
(**a**) Experiment layout: on the floor of an indoor hall, two anchor nodes are located at fixed positions and a mobile node is placed at reference gridpoints in a 5×5 square grid. (**b**) Experiment environment.

In this section, we present experimental evaluation results for the three schemes of the proposed method: MSR1, MSR2, and MSR3, and compare them with the two existing ranging schemes: AltDS-TWR and AltDS-TWR&PR. Experiments were conducted in an indoor hall with line-of-sight between all relevant nodes, as shown in Figure 1. The experimental setup consisted of a master node, two anchor nodes, and a mobile node. Each node is based on Decawave’s DW1000 UWB transceiver [23], which is an IEEE 802.15.4-2011 UWB implementation. Here, we use the master node to command initiation of ranging sessions for each scheme, receive sensing information from anchor and mobile nodes, and log data to a computer via serial UART. The anchor and mobile nodes were all calibrated, respecting the analog delays. The two anchor nodes, A1 and A2, were located 360 cm apart at fixed positions, and the mobile node, M, was placed at predetermined gridpoints in a 5×5 square grid. As shown in Figure 1, each square within the grid was $90\times 90\;{\mathrm{cm}}^{2}$. The coordinates of anchor nodes were A1 =(0cm,0cm) and A2 =(0cm,360cm). Subsequently, coordinates of the reference mobile positions and their corresponding ground-truth ranges from A1 and A2 were computed. For the experiment, we have A1 as an active anchor node, which transmits packet during a ranging session, and A2 as a passive anchor node, which listens to all the packets during a ranging session. During the experiment, ranging for each reference mobile position was conducted 200 times and root-mean-square error (RMSE) of the estimation by each scheme was obtained.

**Figure 2:**
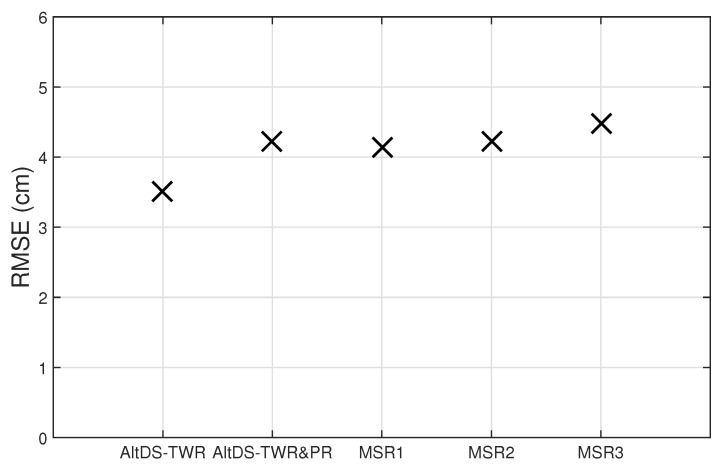
Root-mean-square error (RMSE) of the estimated range versus ranging scheme.

Figure 2 compares RMSE of estimated range by AltDS-TWR, AltDS-TWR&PR, MSR1, MSR2, and MSR3, where the plots show the RMSEs obtained are less than 5cm for all schemes. Note that for the considered experiment scenario, TOFs to both the anchor nodes, A1 and A2, are estimated. Then, the MSE of estimated TOF by the proposed method may be computed using Equation (36) by $\frac{1}{2}\times \sigma_{e}^{2}+\frac{1}{2}\times 2\sigma_{e}^{2}=\frac{3}{2}\sigma_{e}^{2}$, and the RMSE is $\sqrt{\frac{3}{2}}\sigma_{e}$. Recall for the method of AltDS-TWR, the MSE of estimated TOF is $\sigma_{e}^{2}$, and hence the RMSE is $\sigma_{e}$. This is consistent with the result in Figure 2, where the RMSEs by the proposed schemes are approximately one-fifth higher than that by AltDS-TWR. From Figure 2, the RMSE of estimated range by AltDS-TWR is 3.5cm, which is approximately 0.9cm less than that by MSR3 and approximately 0.7cm less than that by the other remaining schemes. The reason for MSR3 to have slightly larger RMSE compared with MSR2 may be the fact found from our experimental data that the standard deviation of the clock-speed ratio estimated in MSR3 is slightly larger. Nonetheless, the overall results indicate that the RMSEs by all the methods are of the same order of magnitude, and hence, all the methods should be applicable to the same accuracy-demanding RTLS applications.

**Figure 3:**
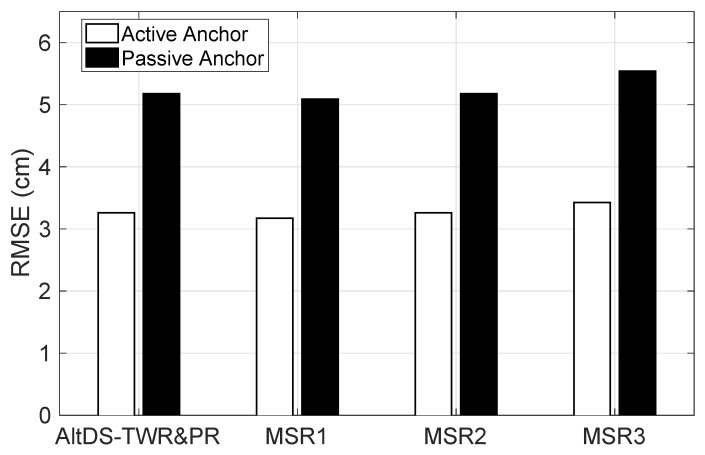
RMSE versus type of involved anchor node for various schemes: alternative double-sided two-way ranging with passive ranging (AltDS-TWR&PR), MSR1, MSR2, and MSR3.

Figure 3 plots the RMSE versus type of involved anchor node, active or passive, for AltDS-TWR&PR, MSR1, MSR2, and MSR3. Note that according to Equation (36), the RMSE of estimated TOF is $\sigma_{e}$ for the active anchor node or $\sqrt{2}\sigma_{e}$ for the passive anchor node. In this regard, the RMSE of estimated range for the passive anchor node A2 should be 1.4 times higher than that for the active anchor node A1. This relation is consistent with the results in Figure 3, where the RMSEs of estimated range for the passive anchor node for all the considered schemes are approximately two-fifth higher than that for the active anchor node. Nonetheless, the RMSEs are of the same order of magnitude, below very few centimeters.

## 4. Further Extensions

The proposed method can be implemented by a variety of schemes, which may be distinguished by the ranging session initiator (who transmits the first packet), the selected node of preferred clock, and the method to determine the clock-speed ratio. An interesting scheme is where an anchor node is both the session initiator and the selected node of the preferred clock, the anchor node periodically initiates ranging sessions, and a mobile node needs to receive the first packet of a session before transmitting a packet into one of several contention slots that follow. This scheme has an interesting advantage in that ranging from multiple mobile nodes to multiple anchor nodes is possible by a single short session.

The proposed method may also be applied in the case that all nodes are mobile and the information of TOFs between the mobile nodes is fused with other sensing information to estimate the node locations [24]. In this case, one session of the proposed method based on Equation (16) can provide one TOF estimate, i.e., ${\hat{T}}_{P(M,A)}$, and a number of TDOA estimates, i.e., ${\hat{T}}_{P(M,X)}-{\hat{T}}_{P(A,X)}$ for *X* not being M or A. It may be noted that the TDOA estimates may be used directly in a sensor fusion scheme, or used for assisting estimation of all required TOFs.

## 5. Conclusions

This paper proposes a method of multiple simultaneous ranging in IR-UWB networks, based on a property of time difference of reception of two packets transmitted from different sources. The proposed method may be considered as a unification of the AltDS-TWR method and a recent asynchronous TDOA method, and a significant extension to both of them. It handles the unwanted clock offsets by the same manner as the two methods, and hence maintains similarly excellent robustness to the clock offsets. Based on an experiment comparing various ranging methods in a line-of-sight environment, the ranging accuracy of the proposed method should also be acceptable for current accuracy-demanding RTLS applications. The proposed method could be much more air efficient, compared with AltDS-TWR and AltDS-TWR&PR, enabling an RTLS to serve significantly more users. Particularly, the MSR1 scheme of the proposed method is most air efficient when a mobile node is designated to be the ranging session initiator, while the MSR3 scheme is most air efficient when an anchor node is designated to be the ranging session initiator.

## Figures and Tables

**Table 1:** Comparison of air time occupancy in wireless networks with *N* number of anchor nodes.

Schemes	Air Time Occupancy	Required Number of
	(Packets)	Packets (for N=4)
AltDS-TWR (w/o combined transmissions)	3N	12
AltDS-TWR (w/combined transmissions)	N+2	6
AltDS-TWR&PR	4	4
MSR1	3	3
MSR2	4	4
MSR3	2	2
